# Zinner Syndrome: An Incidental Finding

**DOI:** 10.7759/cureus.105001

**Published:** 2026-03-10

**Authors:** Pedro Serrano, Pedro Barros, Ana Sofia Moreira, Marco Dores, Aníbal Coutinho

**Affiliations:** 1 Department of Urology, Hospital de Faro, Faro, PRT; 2 Department of Radiology, Hospital de Faro, Faro, PRT

**Keywords:** magnetic resonance imaging, renal agenesis, seminal vesicle cyst, wolffian duct, zinner syndrome

## Abstract

The authors present a case of a 56-year-old man referred to the urology clinic after an incidental finding on abdominal ultrasound. Imaging revealed an oval cystic lesion adjacent to the right seminal vesicle, initially interpreted as a probable atrophic ectopic kidney. Magnetic resonance imaging (MRI) confirmed right renal agenesis, ipsilateral seminal vesicle cyst, and probable ejaculatory duct obstruction. A small intralesional nodular component was identified.

The patient was asymptomatic, with preserved renal function and a prostate-specific antigen of 0.9 ng/mL. A transrectal biopsy was performed due to the suspicious nodule. Histopathological analysis demonstrated seminal vesicle/ejaculatory duct tissue with stromal edema lined by a single-layer cuboidal epithelium (CK7+, 34BE12+, PAX8+, PSA−, CK20−) and low Ki-67 (<1%), with no evidence of malignancy.

Conservative management with imaging surveillance was adopted. Follow-up MRI at six-month intervals showed lesion stability. The patient remains asymptomatic, emphasizing the role of imaging in the diagnosis, assessment, and monitoring of asymptomatic Zinner syndrome.

## Introduction

Zinner syndrome is a rare congenital anomaly of the Wolffian duct, classically defined by the triad of unilateral renal agenesis, ipsilateral ejaculatory duct obstruction, and seminal vesicle cyst formation [1]. Embryologically, abnormal development of the distal mesonephric duct disrupts both renal and seminal tract formation [1,2]. Most cases are diagnosed in the third or fourth decade of life, although some may remain asymptomatic until incidental imaging detection [3].

Clinical manifestations vary widely. Symptomatic patients may present with perineal pain, hematospermia, painful ejaculation, infertility, or lower urinary tract symptoms, while many remain asymptomatic [3-5]. Imaging plays a central role in diagnosis. Ultrasound may suggest congenital anomalies but is limited in anatomical characterization. Magnetic resonance imaging (MRI) provides superior soft-tissue resolution, delineates seminal vesicle cysts and ejaculatory ducts, identifies intralesional nodules, and aids in differentiating cystic lesions from other pelvic masses [1,2].

Management is individualized based on symptoms, cyst size, and reproductive considerations. Asymptomatic patients are typically managed conservatively with periodic imaging surveillance, while intervention is reserved for symptomatic or complicated cases [6]. We report an incidental imaging diagnosis of Zinner syndrome detected on MRI, highlighting the characteristic radiologic findings and their embryologic correlation.

## Case presentation

A 56-year-old male smoker, with a medical history of dyslipidemia and systemic lupus erythematosus under follow-up in an autoimmune disease clinic, was referred to the urology department following an incidental finding on abdominal ultrasound [1,3]. Ultrasound examination revealed an oval cystic structure adjacent to the right lateral aspect of the prostate and seminal vesicle, initially interpreted as a probable atrophic ectopic kidney with dilatation of the collecting system and an 8 mm intralesional nodular component [1,4,5].

The patient was completely asymptomatic, reporting no lower urinary tract symptoms, hematuria, or hematospermia. Laboratory evaluation demonstrated preserved renal function and a prostate-specific antigen level of 0.9 ng/mL [1,3].

**Figure 1:**
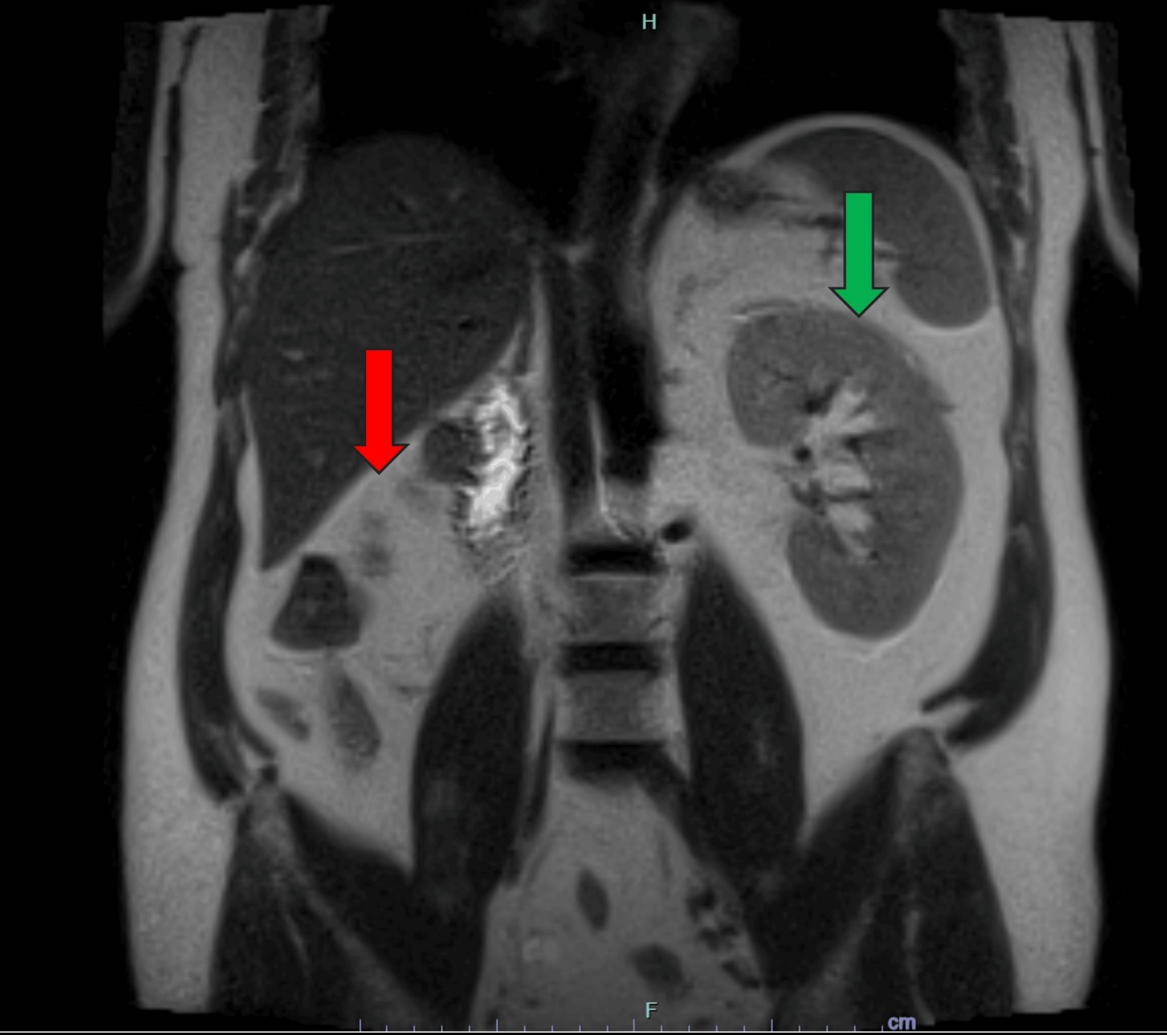
Coronal T2-weighted (T2W) MRI of the upper abdomen demonstrating right renal agenesis (red arrow) and a normal left kidney (green arrow). The image clearly depicts the absence of the right kidney and the normal anatomical appearance of the contralateral kidney. MRI: magnetic resonance imaging

**Figure 2:**
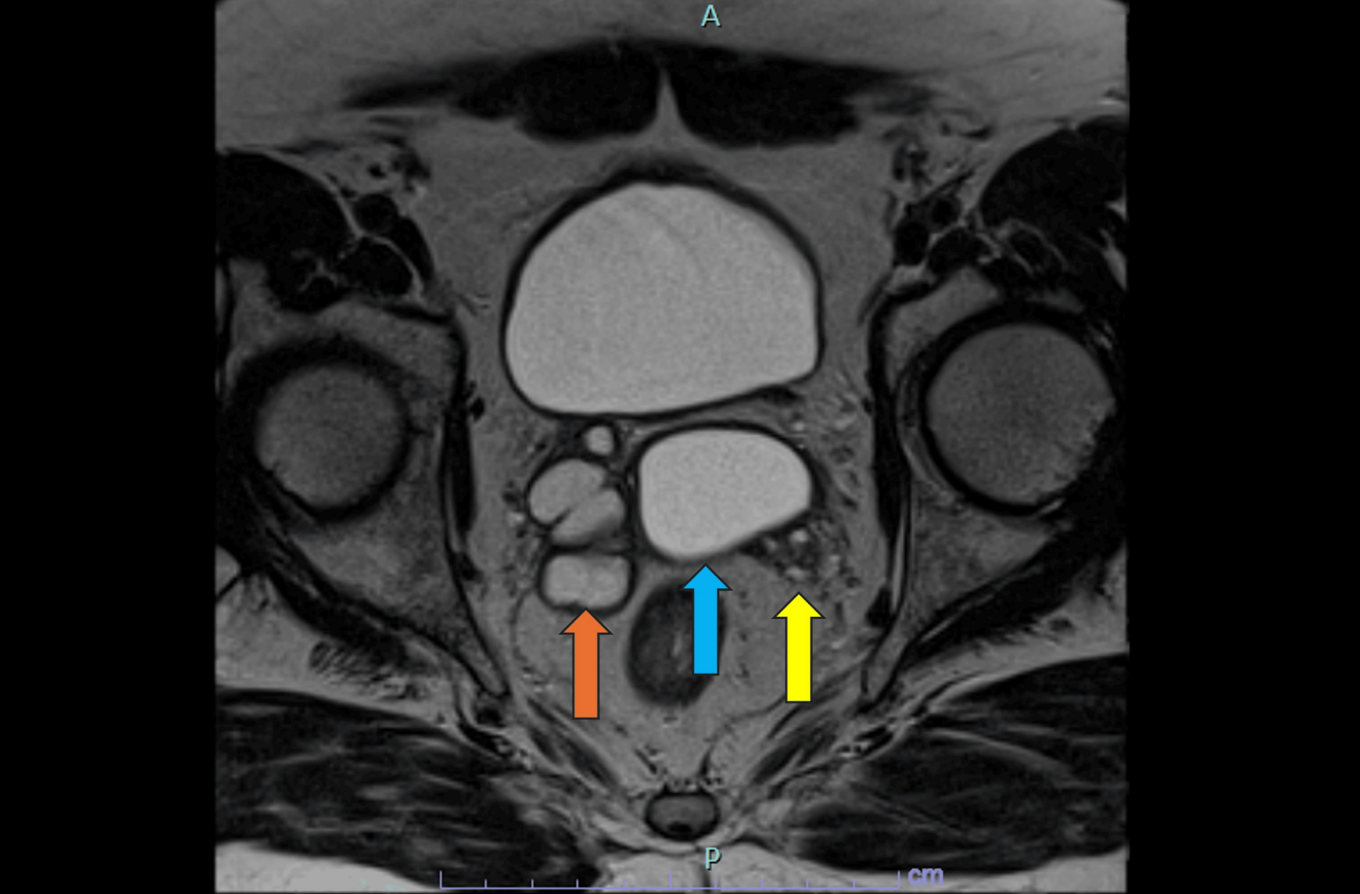
Axial T2-weighted (T2W) MRI of the pelvis showing the ejaculatory duct cyst (blue arrow) and the cystic dilation of the right seminal vesicle (orange arrow). The left seminal vesicle is indicated by the yellow arrow. The cystic right seminal vesicle is ipsilateral to the renal agenesis (see Figure 1), emphasizing the typical triad of Zinner syndrome. MRI: magnetic resonance imaging

**Figure 3:**
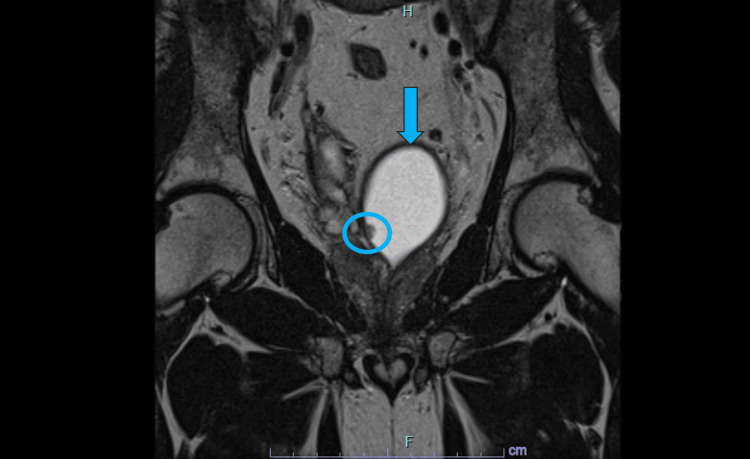
Coronal T2-weighted MRI of the pelvis demonstrating ejaculatory duct obstruction with cyst formation (blue arrow). A small intralesional nodular component is visible along the medial aspect of the cyst (blue circle), which prompted histopathological evaluation. MRI: magnetic resonance imaging

**Figure 4:**
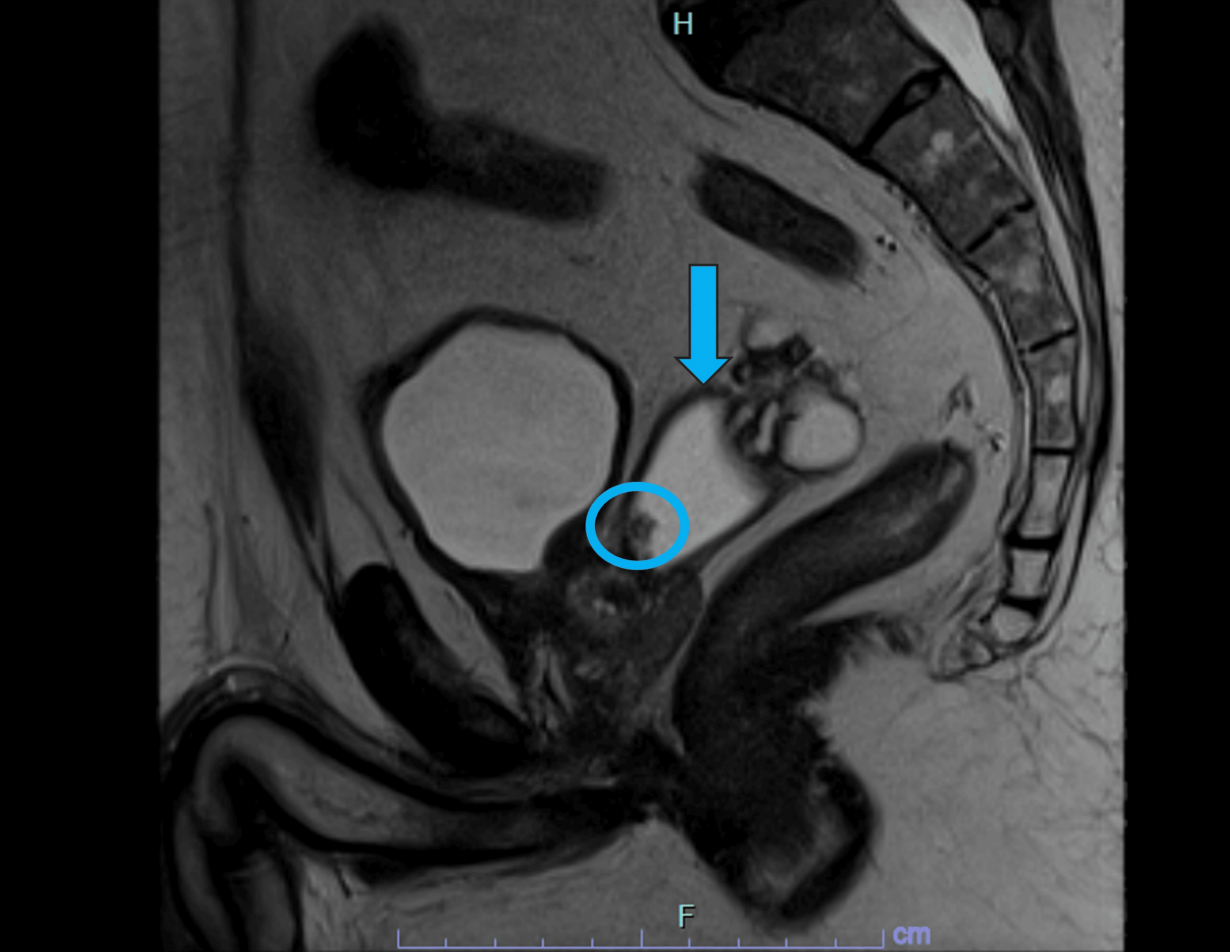
Sagittal T2-weighted (T2W) MRI of the pelvis demonstrating the ejaculatory duct cyst (blue arrow) with a small intralesional solid nodule (blue circle). MRI: magnetic resonance imaging

MRI demonstrated right renal agenesis and a large cystic lesion arising from the region of the ejaculatory ducts at the base of the prostate. The lesion measured approximately 50 × 39 × 28 mm (estimated volume 28 cc) and was associated with cystic dilation of the right seminal vesicle. A small enhancing intralesional nodular component measuring approximately 7 × 4 × 4 mm was identified along the medial inferior aspect of the cyst, adjacent to the ectopic ureteric insertion. No diffusion restriction was observed, likely due to the small size of the lesion (Figures 1-4) [1,2,7]. The imaging findings suggested a congenital anomaly of the mesonephric duct system compatible with Zinner syndrome.

Given the presence of a small enhancing nodular component within the cystic lesion on MRI, histopathological evaluation (transrectal biopsy) was performed to exclude malignancy. Histopathological examination revealed fragments consistent with seminal vesicle/ejaculatory duct tissue showing prominent stromal edema and cystic glandular structures lined by a single-layer cuboidal epithelium. Immunohistochemical staining demonstrated CK7+, 34BE12+, and PAX8 positivity, with negative PSA and CK20 expression. The proliferative index was low (Ki-67 < 1%), and no cytological atypia or features suggestive of malignancy were identified [1,2,4].

A conservative management strategy with imaging surveillance was adopted. Serial MRI at six-month intervals demonstrated dimensional stability of the cystic lesion and nodular component [1,5,8]. The patient remains completely asymptomatic, illustrating that asymptomatic Zinner syndrome with a small nodular component can be safely monitored without immediate intervention [6,9].

## Discussion

Zinner syndrome should be considered in male patients presenting with unilateral renal agenesis and associated pelvic cystic lesions. The increasing availability and routine use of cross-sectional imaging modalities have led to a higher rate of incidental diagnoses, particularly in asymptomatic adult patients [2,5,7]. This trend is consistent with recent reports describing late or incidental detection in patients undergoing imaging for unrelated indications [7].

Clinical presentation varies widely, ranging from infertility, painful ejaculation, and recurrent infections to complete absence of symptoms [4,7]. In contrast to most published cases involving symptomatic younger patients or infertility-related investigations [3,5,9], the present case describes an older, completely asymptomatic patient, highlighting the heterogeneity of disease presentation and reinforcing the importance of individualized management strategies.

Management decisions in Zinner syndrome depend on symptom severity, cyst size, and reproductive considerations. Asymptomatic patients are generally managed safely with conservative imaging surveillance, while surgical intervention is reserved for symptomatic cases, large cysts (commonly >5 cm), or failure of conservative measures [6,8,9]. Reported surgical options include open or laparoscopic excision, transurethral balloon dilatation of the ejaculatory duct, and transrectal ultrasound-guided aspiration, although recurrence after minimally invasive approaches has been described [5,6].

MRI plays a central role in diagnosis and follow-up, providing superior soft-tissue characterization and accurate delineation of seminal vesicle cysts, ejaculatory duct obstruction, and associated intralesional components [1,3,7]. The presence of a small intralesional nodular component, as observed in this case, has been rarely reported and may raise concern for neoplastic transformation, although malignant degeneration remains exceptionally uncommon [6,7]. In such scenarios, MRI features combined with histopathological confirmation are essential to safely exclude malignancy and avoid unnecessary surgical intervention.

This case underscores the value of MRI-guided diagnostic assessment and histological confirmation in selected patients. Similar conservative approaches have been reported in asymptomatic individuals with stable imaging findings and small seminal vesicle cysts, where surgery was avoided unless symptoms developed or lesion progression occurred. Compared with previously published cases, the presence of a small intralesional nodular component in our patient raised concern for possible neoplastic transformation, which prompted biopsy, although malignant degeneration in Zinner syndrome remains exceptionally rare [1-9].

## Conclusions

Zinner syndrome is an uncommon congenital anomaly that should be considered in male patients presenting with unilateral renal agenesis and associated pelvic cystic lesions, including those detected incidentally. MRI plays a pivotal role in diagnosis, characterization, and follow-up, particularly when intralesional components are identified. In asymptomatic patients with stable imaging findings and no evidence of malignancy, conservative management with imaging surveillance represents a safe and appropriate strategy, allowing avoidance of unnecessary surgical intervention while preserving reproductive potential.
